# Microplastics in marine pollution: Oceanic hitchhikers for the global dissemination of antimicrobial-resistant bacteria^☆^

**DOI:** 10.1016/j.onehlt.2025.101056

**Published:** 2025-05-05

**Authors:** Gustavo Queiroga, Felipe Vásquez-Ponce, Thais Martins-Gonçalves, Nilton Lincopan

**Affiliations:** a Department of Clinical and Toxicological Analysis, School of Pharmacy, University of São Paulo, São Paulo, Brazil; b One Health Brazilian Resistance Project (OneBR), Brazil; c Department of Microbiology, Institute of Biomedical Science, University of São Paulo, São Paulo, Brazil; d Antimicrobial Resistance Institute of São Paulo (ARIES), São Paulo, Brazil; e Department of Pathology, School of Veterinary Medicine and Animal Science, University of São Paulo, São Paulo, Brazil

**Keywords:** Antimicrobial resistance, Carbapenemases, Aquatic environments, Plastisphere, One Health

## Abstract

Microplastics (MPs) are globally anthropogenic contaminants of marine environments. Bacteria can colonize MPs forming biofilms that constitute the plastisphere. Carbapenem-resistant bacteria in plastisphere could be a hidden threat for marine life. The role of MPs in the spread of AMR bacteria/genes deserves global investigation.

## Highlights

• Microplastics (MPs) are globally anthropogenic contaminants of marine environments.
• Bacteria can colonize MPs forming biofilms that constitute the plastisphere.
• Carbapenem-resistant bacteria in plastisphere could be a hidden threat for marine life.
• The role of MPs in the spread of AMR bacteria/genes deserves global investigation.

Microplastics (MPs) are commonly described as tiny plastic particles or pieces smaller than 5 mm in diameter, that come from the degradation of plastics, being insoluble in water and ubiquitous in nature, affecting aquatic environments, wildlife, and humans [1]. In this regard, since MPs, biocides (antibiotics, disinfectants), and heavy metals have emerged as environmental contaminants, their interaction could contribute synergistically to development, persistence, transport, and dissemination of antimicrobial resistance at the human-animal-environmental interface, becoming a threat to global health and a One Health challenge that deserves to be examined [2].

**Fig. 1:**
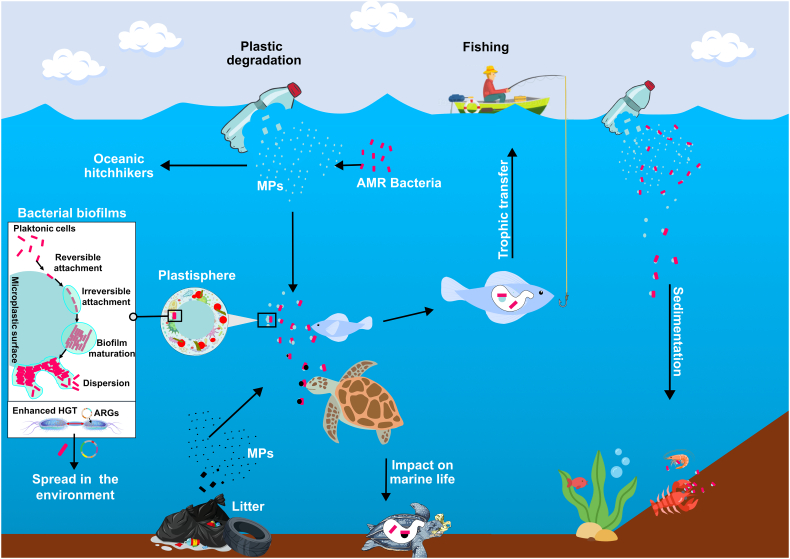
Microplastics in marine pollution as oceanic hitchhikers for the dissemination of antimicrobial-resistant bacteria. Anthropogenically, plastics are discarded in coastal and aquatic environments being degraded through abiotic and physical factors, leading to the fragmentation to smaller particles, designated as microplastics (MPs). Additionally, MPs could be produced from deep-sea litter degradation. The microbial community adheres to the surface of microplastics and colonizes them leading to the formation of the plastisphere. Physico-chemical changes resulting from plastisphere formation can produce an increase of MP density leading to sedimentation. Finally, microplastics carrying antimicrobial-resistant bacteria and resistance genes can spread throughout the aquatic environment impacting marine life and human beings (trophic chain).

In aquatic environments, including coastal waters, MPs can serve as a habitat for microbial life, on which they can form biofilms, acting as a long-distance transport mechanism for human and animal pathogens, and potentially spread antibiotic-resistant bacteria into new areas [3]. Biofilms can develop rapidly on floating MPs changing their density and overall size [4]. The specific interaction between bacterial biofilms and microplastics leads to the formation of the plastisphere, which may persist for a long time, acting as reservoir for various contaminants (*i.e*., heavy metals, biocides, and antibiotics) adsorbed by microplastic biofilms through hydrophobic, electrostatic, and/or hydrogen bonding interactions [4]. While the long half-life, hydrophobicity and rough surface of MPs can effectively improve and enhance the survival of microorganisms under adverse environmental conditions, organic detritus absorbed on MPs contributes to maintenance and proliferation of microbes [3,4]. The plastisphere formation includes: i) coverage of MP surfaces by marine environmental contaminants, which are reversibly and irreversibly adhered by microorganisms; ii) microbial colonization with production of extracellular polymer substances, where short-range forces (*i.e*., ionic bonding, covalent bonding, and hydrogen bonding) associated with irreversible binding contribute to stronger adhesion of microorganisms that lead to the formation of the biofilm; and iii) proliferation of microorganisms on the MPs surface [4] (Fig. 1).

Interestingly, the plastisphere can contribute to the selection and spread of resistant strains, and induction of horizontal gene transfer (HGT) of antibiotic resistance genes (ARGs), where sessile bacteria perform HGT more effectively than planktonic bacteria. Indeed, some studies have shown an increase of HGT in MPs via conjugation [[3], [4], [5], [6]]. Additionally, antimicrobial-resistant bacteria have been reported at concentrations 100–5000 times higher on microplastic surfaces than in the surrounding seawater [7]. Consequently, Gram-negative bacteria categorized within the “critical group” by the World Health Organization (https://www.who.int/publications/i/item/9789240093461), as broad-spectrum cephalosporin-resistant *Escherichia coli* producing extended-spectrum beta-lactamase CTX-M-9, and carbapenem-resistant *Klebsiella pneumoniae* and *K. quasipneumoniae* producing KPC-3 carbapenemases have been isolated from aquatic microplastics [5,6], alerting on a potential vehicle for the dissemination of clinically relevant antibiotic-resistant bacteria, which have expanding beyond hospital walls [8].

In large and populous countries with coastline length and high percentage of the population living in such areas, as Brazil, China, India, and the United States of America (USA), highest per capita plastic consumption and a significant mismanagement of plastic waste have been documented [9]. Specifically in Brazil, a country with the longest inter and subtropical coastline in the world, the anthropogenic impact of coastal pollution has been a critical issue [10]. In this regard, while the persistence of marine litter in the continental slope of some Southern and Southeast states was recently reported [10], cocaine and other illicit drugs have been also detected, impacting sharks inhabiting this area [11]. Unfortunately, in Brazil, marine pollution has also been associated with the dissemination of clinically relevant antimicrobial-resistant bacteria [[12], [13], [14], [15], [16]], transforming the marine ecosystem in a “hotspot” for the emergence, maintenance, and dispersal of ARGs, and antibiotic-resistant microorganisms [12]. In fact, several studies have documented the presence of WHO critical priority carbapenem-resistant bacterial clones in coastal waters, including carbapenemase (KPC-2)-positive *Enterobacter cloacae* sequence type (ST) ST520 (GenBank: MWLI00000000.1), *E. kobei* (GenBank: OR098884.1), *Citrobacter* spp., *Kluvyera* spp., and *Aeromonas* [13], as well as NDM-1-producing *Klebsiella pneumoniae* ST11 [14].

Noteworthy, the antimicrobial resistance problem in the marine environment has impacted the resident fauna of the Atlantic coast of Brazil, becoming a One Health issue [15,16]. In this regard, extended-spectrum beta-lactamase (CTX-M-15)-producing *E. hormaechei* ST114 and *C. freundii* ST265 co-infecting free-living green turtle (*Chelonia mydas*) has been reported [15], whereas more recently NDM-1-producing *C. freundii* ST18 has been isolated from a green sea turtle impacted by plastic pollution [16]. On the other hand, among Gram-positive pathogens, *Enterococcus faecalis* and *E. faecium* resistant to ciprofloxacin, erythromycin, norfloxacin, tetracycline, and/or rifampicin, and carrying *tet(S)*, *tet(M)*, *tet(L)*, *mrsC*, and *erm(B)* resistance genes have been isolated from sea turtles and mammals (*i.e*., common minke whale, humpback whale, and Risso's dolphin) found alive or dead in the southern coast of Brazil [17]. Another critical issue for marine wildlife conservation/preservation, in this country, has been the emergence of infections in migratory Magellanic penguins by polymyxin-resistant *E. coli* belonging to the pandemic ST10, carrying both the mobile colistin resistant gene *mcr-1*, and the *bla*_CTX-M_ ESBL gene [18]. Similar epidemiologic events have also been reported in the USA, with beta-lactamase-producing Enterobacterales (*i.e*., *E. coli*, *K. pneumoniae*, *Citrobacter* spp., and *Enterobacter cloacae* complex) displaying a multidrug-resistant profile to trimethoprim-sulfamethoxazole, tetracycline, chloramphenicol, gentamicin, tobramycin, ciprofloxacin, and fosfomycin; associated with *sul*, *tet(A)*, *strA*, *strB* resistance genes, and mutations in the quinolone resistance-determining regions (QRDR) of *gyrA* [83 (Ser to Leu) and 87 (Asp to Asn)] and *parC* [80 (Ser to Ile)] genes, being isolated from Pinnipeds, such as northern elephant seals, harbor seals, and California sea lions [19]. In brief, the colonization and/or infection by antibiotic-resistant bacteria in marine wildlife could be related with anthropogenic pollution linked to hospital/urban wastewater, MPs, and plastisphere formation, which can then be dispersed throughout an ecosystem [20].

Strategies for tackling antimicrobial resistance caused by MPs pollution should include: i) improve public awareness and restrict the use of microplastics; ii) standardize methodologies and disseminate guidelines for the overall monitoring and assessment of plastic waste and microplastics; and, iii) establish the removal of MPs from wastewater through physical (filtration, magnetic separation, adsorption), chemical (oxidation treatment), and/or biological (biodegradation) methods to prevent infiltration into aquatic environments [21,22].

Finally, despite efforts to combat plastic pollution in marine environments, the legislation developed by countries is often neither comprehensive nor effective [23]. Some countries as Uruguay and Argentina have implemented a national ban on certain single-use plastics [23]. On the other hand, non-governmental organizations (NGOs) have played a mediating role between the government and citizens, working to protect the environment through various awareness and networking activities, collaborating with governments to promote key environmental initiatives, including waste management [23].

In summary, we alert on the potential for MPs to become oceanic hitchhikers for dissemination of clinically relevant antimicrobial-resistant bacteria and their ARGs in marine environments, emphasizing the need for collaborative international efforts to address this global issue and strengthen policy recommendations to control MP contamination.

## CRediT authorship contribution statement

**Gustavo Queiroga:** Writing – review & editing, Writing – original draft, Visualization, Validation, Formal analysis, Conceptualization. **Felipe Vásquez-Ponce:** Writing – review & editing, Writing – original draft, Visualization, Validation, Formal analysis, Conceptualization. **Thais Martins-Gonçalves:** Writing – review & editing, Writing – original draft, Visualization, Validation, Formal analysis, Conceptualization. **Nilton Lincopan:** Writing – review & editing, Writing – original draft, Visualization, Validation, Formal analysis, Conceptualization.

## Declaration of competing interest

The authors declare that they have no known competing financial interests or personal relationships that could have appeared to influence the work reported in this paper.

## Data Availability

No data was used for the research described in the article.
